# 2D ^1^H sLASER Long‐TE and 3D ^31^P Chemical Shift Imaging at 3 T for Monitoring Fasting‐Induced Changes in Brain Tumor Tissue

**DOI:** 10.1002/jmri.29422

**Published:** 2024-05-09

**Authors:** Seyma Alcicek, Iris Divé, Dennis C. Thomas, Vincent Prinz, Marie‐Thérèse Forster, Marcus Czabanka, Katharina J. Weber, Joachim P. Steinbach, Michael W. Ronellenfitsch, Elke Hattingen, Ulrich Pilatus, Katharina J. Wenger

**Affiliations:** ^1^ Institute of Neuroradiology University Hospital Frankfurt, Goethe University Frankfurt/Main Germany; ^2^ University Cancer Center Frankfurt (UCT) Frankfurt/Main Germany; ^3^ Frankfurt Cancer Institute (FCI) Frankfurt/Main Germany; ^4^ German Cancer Research Center (DKFZ) Heidelberg, Germany and German Cancer Consortium (DKTK) Partner Site Frankfurt/Mainz Germany; ^5^ Dr. Senckenberg Institute of Neurooncology University Hospital Frankfurt, Goethe University Frankfurt/Main Germany; ^6^ Center for Personalized Translational Epilepsy Research (CePTER) Goethe‐University Frankfurt Frankfurt/Main Germany; ^7^ Department of Neurosurgery University Hospital Frankfurt, Goethe University Frankfurt/Main Germany; ^8^ Institute of Neurology (Edinger‐Institute) University Hospital Frankfurt, Goethe University Frankfurt/Main Germany

**Keywords:** MR spectroscopy, ^1^H sLASER long‐TE, glioma, fasting, ketone bodies

## Abstract

**Background:**

Emerging evidence suggests that fasting could play a key role in cancer treatment. Its metabolic effects on gliomas require further investigation.

**Purpose:**

To design a multi‐voxel ^1^H/^31^P MR‐spectroscopic imaging (MRSI) protocol for noninvasive metabolic monitoring of cerebral, fasting‐induced changes on an individual patient/tumor level, and to assess its technical reliability/reproducibility.

**Study Type:**

Prospective.

**Population:**

MRS phantom. Twenty‐two patients (mean age = 61, 6 female) with suspected WHO grade II‐IV glioma examined before and after 72‐hour‐fasting prior to biopsy/resection.

**Field Strength/Sequence:**

3‐T, ^1^H decoupled 3D ^31^P MRSI, 2D ^1^H sLASER MRSI at an echo time of 144 msec, 2D ^1^H MRSI (as water reference), T1‐weighted, T1‐weighted contrast‐enhanced, T2‐weighted, and FLAIR. sLASER and PRESS sequences were used for phantom measurements.

**Assessment:**

Phantom measurements and spectral simulations were performed with various echo‐times for protocol optimization. In vivo spectral analyses were conducted using LCModel and AMARES, obtaining quality/fitting parameters (linewidth, signal‐to‐noise‐ratio, and uncertainty measures of fitting) and metabolite intensities. The volume of glioma sub‐regions was calculated and correlated with MRS findings. Ex‐vivo spectra of necrotic tumor tissues were obtained using high‐resolution magic‐angle spinning (HR‐MAS) technique.

**Statistical Tests:**

Wilcoxon signed‐rank test, Bland–Altman plots, and coefficient of variation were used for repeatability analysis of quality/fitting parameters and metabolite concentrations. Spearman *ρ* correlation for the concentration of ketone bodies with volumes of glioma sub‐regions was determined. A *P*‐value <0.05 was considered statistically significant.

**Results:**

^1^H and ^31^P repeatability measures were highly consistent between the two sessions. β‐hydroxybutyrate and acetoacetate were detectable (fitting‐uncertainty <50%) in glioma sub‐regions of all patients who completed the 72‐hour‐fasting cycle. β‐hydroxybutyrate accumulation was significantly correlated with the necrotic/non‐enhancing tumor core volume (*ρ* = 0.81) and validated using ex‐vivo ^1^H HR‐MAS.

**Data Conclusion:**

We propose a comprehensive MRS protocol that may be used for monitoring cerebral, fasting‐induced changes in patients with glioma.

**Evidence Level:**

1

**Technical Efficacy:**

Stage 4

Key to the integration of nutritional interventions into cancer treatment is the observation that healthy brain cells and tumor cells appear to differ in their ability to adapt to a diet‐induced nutrient‐deprivation.^1^ Under normal physiological conditions, neurons and glial cells utilize glucose oxidation as a primary energy source.^2^ Glycolysis and respiration remain tightly linked here, resulting in efficient adenosine triphosphate production with little lactic acid production.^2^ When glucose availability is limited, they enter a self‐maintenance mode and switch to alternative catabolic pathways, such as the oxidation of fatty acids or ketone bodies (KBs).^3^ Many tumor cells, such as those in glioblastoma, bear oncogenetic mutations that promote glucose utilization and the Warburg effect.^4^ The rate of glycolysis and lactate (Lac) production in the cytosol are therefore dramatically increased.^5^ What happens when glucose availability is limited is equivocal. Not only are there multiple molecular subtypes of glioblastoma^6^ that seem to have different degrees of metabolic plasticity,^1^, ^2^, ^7^, ^8^ but there is also a high degree of intratumoral heterogeneity with regions of relative hypoxia that can enforce metabolic switches.^9^

A better understanding of diet‐induced changes in tumor tissue on an individual patient/tumor level is therefore crucial to successfully incorporate dietary interventions into current treatment options. In consequence, we took a step back from combination therapies and initiated the open‐label ERGO3 trial (Characterization of Metabolic Changes in the Glioma Tumor Tissue Induced by Transient Fasting; ClinicalTrials.gov Identifier: NCT04461938). In this single‐group study, patients with MRI‐suspected glioma complete one fasting cycle of 72 hours prior to tumor biopsy/resection. In lieu of repeated biopsies and their bias due to warm and cold ischemia time when monitoring fasting‐induced cerebral biochemical changes, we introduced in vivo MR‐spectroscopy (MRS).^10^ The goal was to account for key metabolites related to energy metabolism, tumor heterogeneity, and normal‐appearing brain regions.

Broadly speaking, levels of KBs, Lac, and several other brain metabolites can be determined using proton MRS.^11^ Specifically, KBs should become MRS‐detectable during periods of fasting or ketogenic diets due to their accumulation in the brain in low millimolar concentrations. Accordingly, there are recent reports of acetone (Ace) detection in tumor tissue of glioma patients employing ^1^H MR single‐voxel spectroscopy^11^, ^12^ and ^1^H chemical shift imaging (CSI)^10^ at short‐echo times (TEs, 30–40 msec). The detection of the other KBs, acetoacetate (AcAc), and β‐hydroxybutyrate (β‐OHB) can be more challenging due to their lower signal amplitude caused by fewer number of protons per peak and J‐splitting, respectively.^10^, ^12^ The well‐known advantage of a short TE is to obtain a higher signal‐to‐noise ratio (SNR) due to a reduced T_2_ relaxation and J‐coupling‐induced dephasing. However, the analysis of short‐TE spectra remains a challenge because of a broad baseline originating from cellular macromolecules and lipids, which can be modulated in different tissue types. Especially in tumor tissue, the presence of mobile lipids or pathologically altered macromolecules complicates the baseline modeling further.^13^

At long TEs (>100 msec) macromolecules and lipids with a short T_2_ relaxation time are suppressed improving the spectral fitting for the quantification of metabolites.^14^ In particular, a TE of 144 msec has been suggested for the accurate quantification of Lac, even though there are some pitfalls at 3 T.^15^ A rather flat baseline and the inverted spectral pattern by J‐modulation allow for the differentiation of the corresponding peak at 1.31 ppm and broad lipid peaks arising at the 0.9–1.3 ppm range.^16^ Due to its molecular similarity with Lac (i.e., both consisting of J‐coupled methyl groups), the detection of β‐OHB is improved at long TE as well.^17^ This has been demonstrated by Wootton‐Gorges et al using a point‐resolved spectroscopic (PRESS) sequence at 1.5 T.^17^ However, PRESS localization has a major shortcoming: the chemical‐shift displacement error which hampers accurate metabolite quantification.^15^ The limitation can be overcome with the use of a semi localization by adiabatic selective refocusing (sLASER) sequence where refocusing is achieved by high‐bandwidth adiabatic radiofrequency pulses (>5 kHz), which are combined with outer volume suppression modules.^18^ In brief, sLASER provides sharp slice‐selection profiles, suppresses anomalous J‐evolution, reduces sensitivity to B1 inhomogeneities, and prolongs the apparent T_2_ relaxation times of metabolites.^16^

The combination of ^1^H with ^31^P MRSI can provide complementary biochemical information, particularly, on the cellular energy metabolism (linked to the ratio of adenosinetriphosphate/phosphocreatine [ATP/PCr]), hypoxic physiochemical status (derived from intracellular pH calculated using the chemical shift of endogenous inorganic phosphate), and the rate of membrane synthesis or metabolic turnover (associated with the ratio of phosphomonoester/phosphodiesters [PME/PDE]).^19^ Hence, designing a multi‐voxel, multi‐nuclei (^1^H/^31^P) MRSI protocol with fully automated segmentation of glioma sub‐regions^20^ would be highly useful for monitoring fasting‐induced changes.

Against this background, we aimed to investigate the feasibility, reliability, and repeatability of such a protocol. Furthermore, we aimed to validate in vivo findings using ex vivo high‐resolution magic‐angle spinning (HR‐MAS) NMR.

## Materials and Methods

The study protocol was approved by the local institutional review board (Ethics Committee, University Hospital Frankfurt, Germany, Project number 19‐453), and written informed consent was obtained from the patients.

### Simulations and Phantom Experiments in Preparation of the Clinical Study

^1^H spectra for AcAc, Ace, Lac, and β‐OHB were simulated for the sLASER pulse sequence at TEs = 40, 80, 120, 144, and 288 msec as described below. The T_2_ decay was neglected in the simulation. A 1‐liter bottle phantom was prepared with 5 mmol/L Lac, 5 mmol/L β‐OHB, AcAc, and Ace in phosphate‐buffered saline (pH = 7.3). Due to the non‐enzymatic degradation of AcAc and rapid vaporization of acetone, their concentrations are not specified here. The phantom was placed in the center of the 20‐channel ^1^H head coil. ^1^H spectra of the phantom were acquired using CSI sLASER and CSI PRESS sequences at TE = 40, 80, 120, 144, and 288 msec.

### ERGO3 Study Design

The ERGO3 study was conducted at a tertiary care hospital with a key focus on oncology. It was a prospective, single group study investigating the effects of one 72‐hour‐fasting cycle on glioma tumor tissue. Patients over 18 years of age with MRI‐suspected glioma World Health Organization (WHO) grade II, III, or IV (at the time of study initiation according to the 2016 WHO Classification of Tumors of the Central Nervous System (CNS)^21^) and a recommendation for biopsy/resection were eligible. The 72‐hour‐fasting cycle was followed by tumor biopsy/resection.

### Laboratory Testing

Patient blood samples were analyzed using the Precision Xceed system with blood β‐ketone test strips with a detection limit of 1 mmol/l (Abbott, Chicago, IL, USA).

### MRSI Study Protocol

All patients had undergone routine brain tumor imaging as part of the trial screening process with T1‐weighted (T1W), T1W contrast‐enhanced, T2‐weighted (T2W), and fluid‐attenuated inversion recovery (FLAIR) images available for analysis.

In addition, an extended MRS protocol was performed on another clinical 3‐T MR scanner (MAGNETOM Prisma (VE11C), Siemens Healthineers, Erlangen, Germany) comprising two sessions. Session 1 (S‐1) was conducted as a baseline prior to the 72‐hour‐fasting cycle and Session 2 (S‐2) after the completion of the fasting cycle, prior to biopsy/resection. For each session, measurements were carried out with two different coils: a double‐tuned ^1^H/^31^P volume head coil (RAPID Biomedical GmbH, Rimpar, Germany) and a 20‐channel ^1^H head coil (Siemens Healthineers, Erlangen, Germany) to obtain optimal results for the respective nuclei. The protocol included three‐dimensional (3D)‐T1W and two‐dimensional (2D)‐T2W reference images as well as ^1^H decoupled 3D ^31^P FID CSI, 2D ^1^H sLASER CSI at a TE of 144 msec. In addition, B1 maps^22^ and 2D ^1^H FID CSI sequences with 2° flip angle without water suppression (i.e., as water reference) were recorded for absolute quantification of metabolite concentrations. For the 2D ^1^H sLASER CSI sequence, a transversal slice was positioned to cover the tumor and contralateral normal‐appearing white matter (NAWM). The 3D ^31^P FID CSI sequence was acquired with weighted circular phase encoding while WALTZ4 ^1^H decoupling was applied. The total examination time was ∼40 minutes. Protocol details are listed in Table 1.

**TABLE 1:** MRS Sequence Protocol

Pulse Sequence	3D ^31^P FID CSI	2D ^1^H sLASER CSI	2D ^1^H FID CSI	^1^H sLASER SVS
Echo time	2.3 msec^a^	144 msec	2.3 msec^a^	144 msec
Repetition time	2000 msec	2000 msec	200 msec	2000 msec
Flip angle	60°	90°	2°	90°
Slice thickness	25 mm^b^ interpolated to 12.5 mm	12 mm	25 mm	15 mm
Matrix size and field of view	8 × 8 × 8 at 240 × 240 × 200 mm^3^ interpolated to 16 × 16 × 16	20 × 20 at 240 × 240 mm^2^ interpolated to 40 × 40	20 × 20 at 240 × 240 mm^2^ interpolated to 40 × 40	‐
Voxel size	15 × 15 × 12.5 mm^3^	6 × 6 × 12 mm^3^	6 × 6 × 25 mm^3^	20 × 20 × (15–20) mm^3^
Vector size	1024	1024	512	2048
Bandwidth	2000 Hz	2000 Hz	5000 Hz	2000 Hz
Acquisition time	10:44 m	10:52 m	00:51 m	2:28 m
Number of averages	10	2	1	128
Water suppression	‐	CHESS	None	VAPOR

Each session consisted of two parts: In the first part, a double‐tuned ^1^H/^31^P volume head coil was used to record a 2D T2‐weighted image turbo spin‐echo sequence in the axial plane (2.5 minutes), a 3D T1‐weighted image gradient echo sequence (4 minutes), and a ^1^H decoupled 3D ^31^P FID CSI sequence. In the second part of the session, a 20‐channel ^1^H head coil was used to again record a 2D T2‐weighted image turbo spin‐echo sequence in the axial plane (2.5 minutes), a 3D T1‐weighted image gradient echo sequence (4 minutes), and a 2D ^1^H CSI with sLASER recording the spin echo at TE 144 msec. FID = free induction decay; CSI = chemical shift imaging; sLASER = semi localization by adiabatic selective refocusing; SVS = single voxel spectroscopy; VAPOR = VAriable Power radiofrequency pulses with Optimized Relaxation delays; CHESS = chemical shift selective.

^a^ Delay between excitation and data acquisition.

^b^ Nominal (before interpolation).

In some cases, the CSI spectra were of poor quality due to insufficient B0 static field homogeneity achieved by the vendor‐based shimming routine, which was based on a 3D B0 field map (GRE‐SHIM). Frequently, these tumors were located unfavorably for MRS studies, e.g., in temporopolar region. For such cases, to obtain basic information on tumor metabolism, we switched to single voxel spectroscopy with voxels placed in the solid tumor area and the contralateral NAWM.

### Data Processing and Metabolite Quantification

Registration of the multimodal spectroscopic data to 3D‐anatomical data was performed with an in‐house software tool, which was scripted in MatLab (version R2012b; The Mathworks Inc., Natick, MA, USA). A graphical user interface implemented in this tool allowed the selection of voxels from the entire spectroscopic dataset using resliced 3D‐T1W and 2D‐T2W reference images and a CSI grid overlay.

LCModel^23^ was used for the ^1^H spectral analysis. Metabolite signals for the basis set were simulated for the sLASER pulse sequence at TE = 144 msec with prior knowledge of chemical shift and J‐coupling,^24^, ^25^ using the jMRUI plug‐in NMR‐ScopeB (version 6.0, available at http://www.jmrui.eu). The basis set included spectra of 2‐hydroxyglutarate, N‐acetylaspartate (NAA), N‐acetylaspartylglutamate, choline (Cho), creatine (Cr), γ‐aminobutyric acid, glutamate, glutamine, myo‐inositol, glutathione, glycine, alanine, Lac, β‐OHB, AcAc, and Ace. Macromolecules were not modeled. The quality of the fitted data was evaluated by applying the following rejection criteria: metabolite linewidth (full spectral linewidth at the half amplitude of maximum signal; FWHM) >0.1 ppm, existing artifacts, and the highest metabolite SNR <3. Additional rejection thresholds for Cramer‐Rao lower bounds (CRLBs) of metabolite fits were defined^26^, ^27^ as <10% for total Cho, total Cr, and total NAA (i.e., NAA + N‐acetylaspartylglutamate); <25% for Glx (i.e., glutamate + glutamine); <40% for Lac and myo‐inositol; <80% for KBs (β‐OHB, Ace, and AcAc). These thresholds take into account metabolite levels reported in the literature.^19^ Furthermore, CRLBs represent the uncertainty limit for unbiased estimators and were obtained from MRS model fitting of the data.

Metabolite signal intensities were corrected for T_1_ and T_2_ relaxation using previously recorded relaxation times.^28^, ^29^, ^30^ For β‐OHB, Ace, and AcAc, relaxation times were assumed to be the same as for NAA. Quantification of absolute metabolite concentrations was performed based on the tissue water contents measured by the 2D 1H FID CSI pulse sequence (no water suppression) considering the B1 inhomogeneity.^31^ Details can be found in the supplement.

For the assessment of the reliability and reproducibility of our measurements, four ^1^H CSI voxels were selected from the solid tumor area and the contralateral NAWM. The consistent voxel locations between sessions were achieved using anatomical landmarks. The reliability of our quantification estimates in ^1^H MRS measurements was assessed by examining metabolite linewidth and SNR along with CRLBs. The SNR was calculated by dividing the maximum peak amplitude in the baseline‐corrected spectrum by the noise level obtained from the residual.

In the evaluation of the 3D ^31^P CSI data, one voxel from the tumor area and another voxel from the contralateral NAWM area were chosen. Each voxel location corresponded to the location of the selected voxels from ^1^H‐CSI data (due to the smaller voxel size in ^1^H‐CSI) or a single voxel from the ^1^H‐SVS studies. ^31^P spectra were analyzed with jMRUI using the Advanced Method for Accurate, Robust and Efficient Spectral fitting (AMARES) algorithm.^32^ The signal from phosphocreatine was first adjusted to 0 ppm and a spectral fitting model composed of 15 exponentially decaying sinusoids for metabolites phosphocreatine, phosphocholine, phosphoethanolamine, glycerophosphoethanolamine, glycerophosphocholine, adenosine triphosphate, inorganic phosphate, and macromolecules was used as described in a previous publication.^33^ Moreover, ^31^P spectral fitting was evaluated visually by confirming the accurate assignment of all signals and the existence of a flat residual line with equal distribution of noise. The quality of our ^31^P MRS measurements was assessed by examining metabolite linewidth, SNR, and relative SD (SD/mean × 100%) of spectral fitting. In addition, we analyzed the repeatability between the two sessions using the above‐mentioned measures. Since there is no prior hypothesis for a ^31^P metabolite that is not affected by the intervention, the repeatability analysis for ^31^P metabolite ratios was not performed.

The Brain Tumor Segmentation (BraTS) Toolkit^20^ was used for fully automated segmentations of glioma sub‐regions. The segmentation was based on co‐registered MRI data acquired during the routine brain tumor imaging protocol as part of the trial screening process. Three classes were obtained: edema, necrotic region/non‐enhancing tumor core, and contrast‐enhancing tumor. The results were visually verified by a neuroradiologist (KJW) with 8 years of experience. ITK‐SNAP software (version 3.8.0; www.itksnap.org) was used to calculate volumes of the segmented glioma sub‐regions.^34^

### Ex Vivo HR‐MAS NMR Spectroscopy

After 72 hours of fasting, patients underwent surgical resection of tumor tissue. Resected specimens were snap‐frozen in liquid nitrogen and stored at −80°C until the spectroscopic analysis. Samples from areas with specific interest regarding the in vivo MRS results (e.g., necrosis) were taken using a biopsy punch. They were placed into inserts (sample volume of 25 μL) designed to fit into a 4 mm MAS rotor.

The ^1^H HR‐MAS spectra were recorded at 0°C using a Bruker 600 MHz AVANCE NEO equipped with a 4 mm HX probe (Bruker, Billerica, MA, USA). All samples were spun at 5 kHz. A Carr–Purcell–Meilboom–Gill (CPMG) multi spin‐echo pulse sequence with water presaturation was applied. Following 132 msec CPMG time, data was acquired for 400 msec. Overall, 64–128 transients were accumulated for each sample.

### Statistical Analysis

The statistical analysis was performed using OriginPro software (version 2020; OriginLab Corp., Northampton, MA, USA). Due to non‐normal distribution of continuous variables, assessed using Shapiro–Wilk test, a Wilcoxon signed‐rank test as a paired, non‐parametric statistical hypothesis test was used to compare the metabolite linewidth, SNR, as well as uncertainty measures of spectral fitting at baseline and after 72 hours of fasting within tumor tissue and contralateral NAWM. The same test and Bland–Altman plots were used to compare the concentrations of standard metabolites that were not expected to change during the intervention. Bland–Altman bias (the mean difference) between sessions was calculated for each metabolite concentration in tumor. The variability in ^1^H metabolite concentrations and CRLBs between sessions was also assessed with the coefficient of variation (CV) defined as the ratio of the SD to the mean. A mean CV was calculated by averaging CV values of all patient data. Spearman *ρ* correlation coefficients were determined between the concentration of β‐OHB and AcAc in tumor and the volume of necrotic/non‐enhancing tumor core and contrast‐enhancing tumor. A *P*‐value <0.05 was considered statistically significant.

## Results

### Simulations and Phantom Studies

**FIGURE 1:**
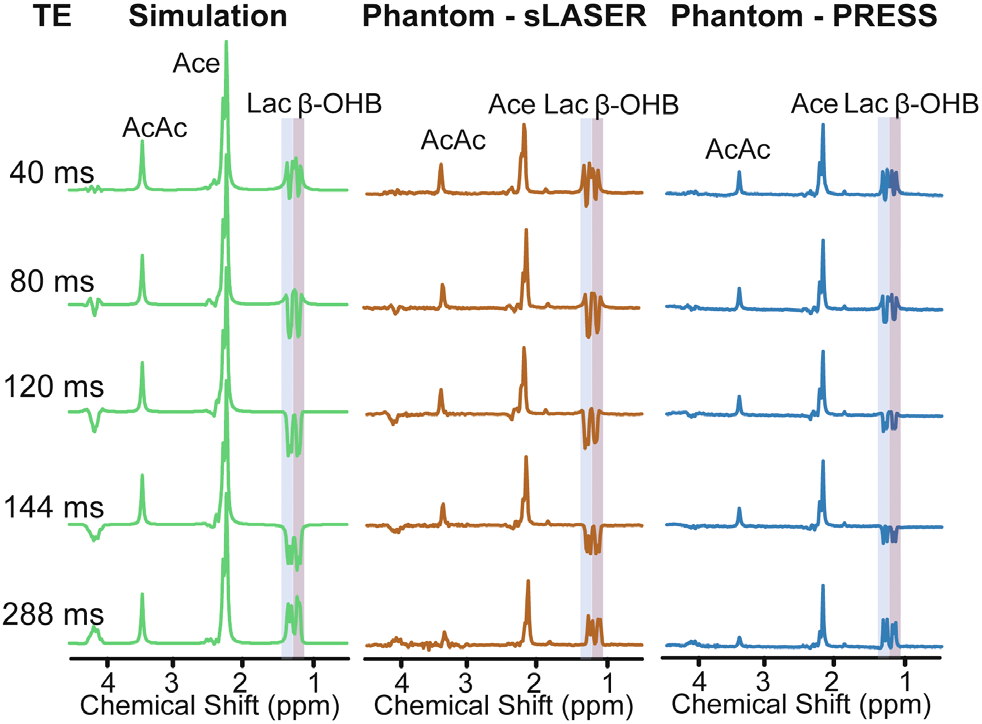
(Left) Simulated spectra of Ace, AcAc, β‐OHB, and Lac for equal concentration at various TEs (40–144 msec) for ^1^H sLASER CSI. Experimental spectra obtained from (Middle) ^1^H sLASER CSI and (Right) ^1^H PRESS CSI measurements of a phantom containing Ace, AcAc, β‐OHB, and Lac.

Simulated spectral patterns of β‐OHB and Lac peaks as a function of sLASER TE (40–288 msec) are demonstrated in Fig. 1 with congruent experimental spectra obtained from phantom measurements using sLASER and PRESS sequences. The doublet peaks originating from methyl groups in β‐OHB and Lac emerged at 1.19 and 1.31 ppm, with their respective 6.3 and 7 Hz J‐coupling constants. At TE = 144 msec, i.e., TE equal to 1/J of Lac, the Lac peak presented as a fully inverted doublet and the β‐OHB peak showed a similar pattern. The β‐OHB doublet was obtained fully inverted at longer TE (≈160 msec) due to a larger J‐coupling constant. For in vivo data, this further increase in TE caused an additional loss of metabolite signals due to T_2_ decay, without improvement in β‐OHB detection, thus a TE of 144 msec was used for ^1^H CSI measurements in the proposed study protocol.

Due to the chemical shift displacement error, the intensities of Lac and β‐OHB peaks obtained with PRESS in the phantom study were lower at TE of 144 msec compared to 288 msec. As demonstrated in Fig. 1, this was not observed in the measurements performed with sLASER localization.

### Patient Characteristics ERGO 3 Study

Of 30 patients recruited for the clinical trial, due to consecutive scanner availability, 22 patients (16 males, 6 females) were examined with our comprehensive MRS protocol at baseline and after 72 hours of fasting. According to the 2021 WHO Classification of Tumors of the CNS, one of these patients was diagnosed with metastases to the CNS (adenocarcinoma) and 21 patients were diagnosed with adult‐type diffuse gliomas WHO CNS grades 2–4.

Overall, 9 patients were excluded from the analysis: the patient diagnosed with a secondary brain tumor, 2 patients who did not complete the required 72 hours of fasting and 6 patients with insufficient spectral quality of the standard protocol. These six patients suffered from tumors located in the temporal lobes. In their ^1^H CSI examinations, an extensive line broadening was observed (>0.1 ppm) due to insufficient B_0_ static field homogeneity caused by magnetic susceptibility gradients. In four of those patients, additional ^1^H sLASER SVS measurements were performed instead (see supplement for details on ^1^H sLASER SVS data). Another patient was excluded from the repeatability analyses of 3D ^31^P CSI due to a failed baseline measurement (hardware problems).

### Reproducibility of ^1^H Spectral Fitting/Quality and Quantification Estimates

**FIGURE 2:**
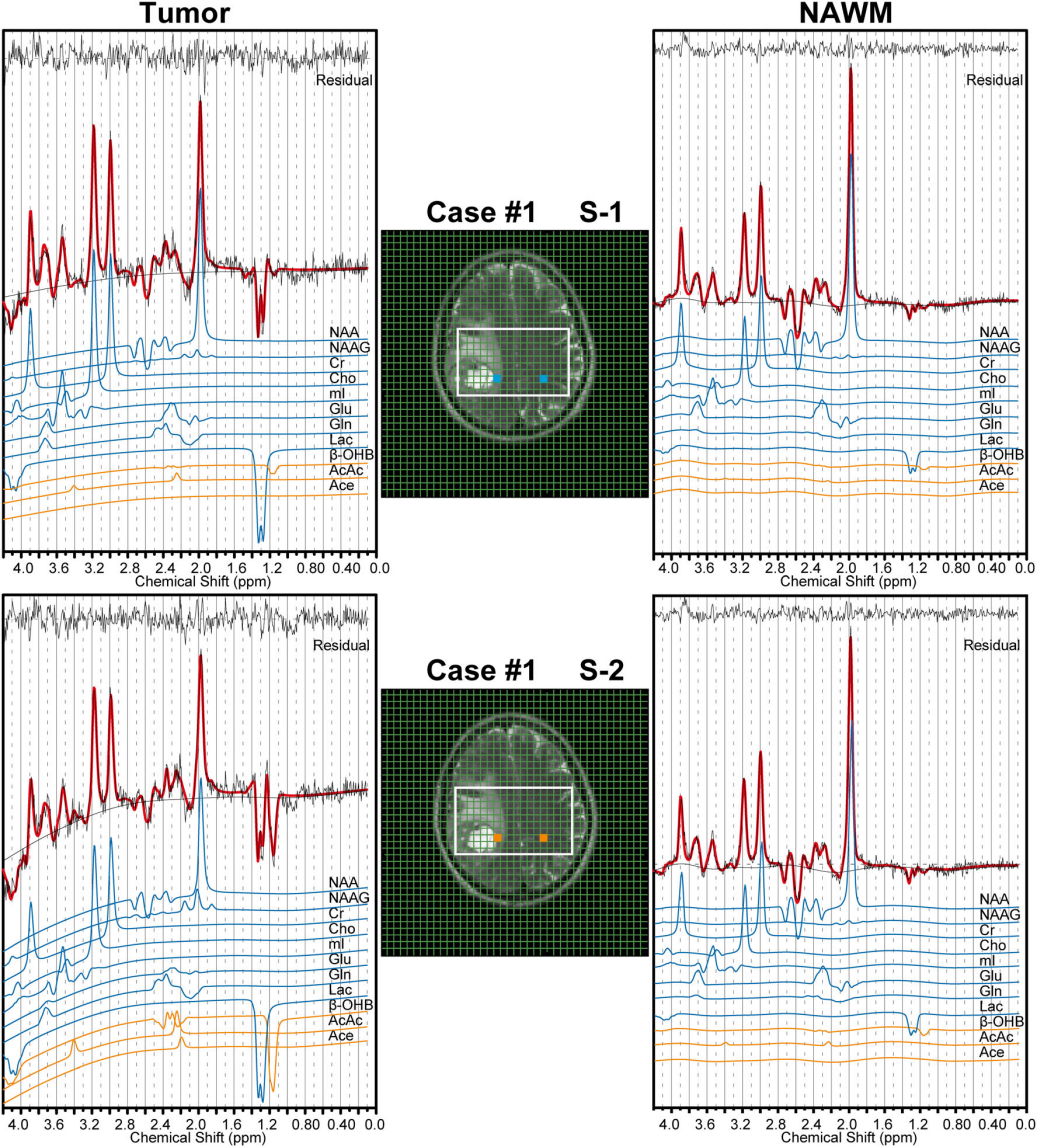
An example analysis of ^1^H CSI data at baseline (S‐1) and after 72 hours of fasting (S‐2) using LCModel with simulated basis data for KBs and standard metabolites. Blue (S‐1) and orange (S‐2) boxes indicate voxel positioning on T2W images. The original signal is presented in black, and the LCModel fit in red. For the standard metabolites and KBs, the individual fitting lines (blue and orange, respectively) are shown. NAAG = N‐acetylaspartylglutamate; mI = myo‐inositol; Glu = glutamate; Gln = glutamine.

Representative ^1^H MRS spectra for a patient and their LCModel fit for KBs (β‐OHB, AcAc, and Ace) and standard cerebral metabolites are shown in Fig. 2. Voxels for both tumor and contralateral NAWM were selected at baseline and after 72 hours of fasting.

**FIGURE 3:**
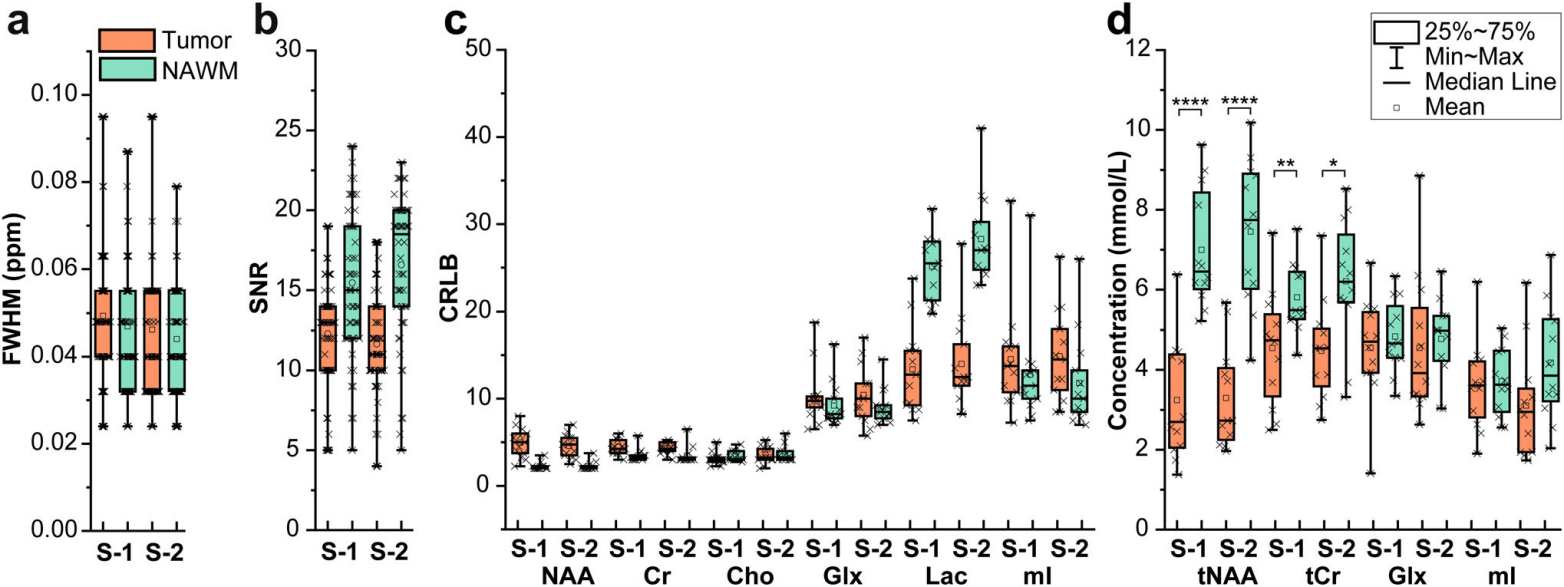
(**a**, **b**) Box plots of LCModel estimated FWHM and SNR of the spectra from the tumor and NAWM at baseline (S‐1) and after 72 hours of fasting (S‐2) that were acquired at 3 T with long (144 msec) TE CSI. (**c**) CRLB comparison of metabolites for the tumor tissue and contralateral NAWM at two sessions. (**d**) Concentration of metabolites that were not expected to differ significantly after dietary period are shown for both sessions. As expected, total NAA and total Cr were found to be significantly lower in the tumor area compared to the contralateral NAWM both at baseline and after 72 hours of fasting. For statistical analysis, a Wilcoxon signed‐rank test as a paired, non‐parametric statistical hypothesis test was used. Results were considered significant at *P < 0.05; **P < 0.01; ***P < 0.001; ****P < 0.001. Box‐and‐whisker plots are presented with minimum, maximum, interquartile ranges (25th and 75th percentiles), and median. tCho = total Cho; tCr = total Cr; tNAA = total NAA; mI = myo‐inositol.

The calculated spectral and fitting/quality parameters (i.e., FWHM, SNR, and CRLBs), as well as concentrations of standard metabolites, that were not expected to change during the intervention, are summarized in Fig. 3.

The FWHM values extracted from both tumor and NAWM spectra were consistent between the two sessions (S‐1 vs. S‐2; *P* = 0.10, *P* = 0.08, from tumor and NAWM spectra, respectively) and outliers were below 0.1 ppm. Likewise, we observed consistent SNR (*P* = 0.42). For both sessions, a lower SNR was observed in tumor spectra compared to NAWM spectra, as the result of a lower intra‐tumoral concentration. Appropriately, CRLBs calculated from tumor (first *P*‐value) and NAWM (second *P*‐value) spectra for NAA (*P* = 0.32, *P* = 1.00), Cr (*P* = 0.62, *P* = 0.86), Cho (*P* = 0.21, *P* = 0.22), Glx (*P* = 0.95, *P* = 0.88), and myo‐inositol (*P* = 0.72, *P* = 0.20) were consistent between the two sessions as well. The significantly higher Lac concentration in tumor tissue compared to NAWM (at baseline 4.92 ± 2.06 mM to 1.80 ± 0.98 mM) resulted in better spectral fitting, i.e., lower CRLBs, demonstrating the relationship between metabolite concentrations and CRLBs. Between‐sessions mean CVs (%) of metabolite concentrations and CRLBs calculated for all patients are summarized in Table 2. The values of mean CV for CRLBs and metabolite in NAWM and tumor were overall less than 16%.

**TABLE 2:** Between‐Sessions Mean Coefficient of Variations (CVs) of Metabolite Concentrations and Cramer‐Rao Lower Bounds (CRLBs) Calculated by Averaging CVs of All Patient Data

	Region	tNAA	tCr	tCho	Glx	Lac	mI
CRLB (%)	NAWM	3.66	4.35	5.56	6.99	8.05	12.22
	Tumor	11.75	6.67	9.21	7.33	6.63	14.02
Concentration (%)	NAWM	10.65	10.34	‐	9.06	‐	12.76
	Tumor	16.23	12.48	‐	12.81	‐	16.42

Concentrations of metabolites that were not expected to differ significantly after dietary period are shown. tNAA = total NAA; tCr = total Cr; tCho = total Cho; Glx = glutamate + glutamine; Lac = lactate; mI = myo‐inositol; NAWM = normal‐appearing white matter.

**FIGURE 4:**
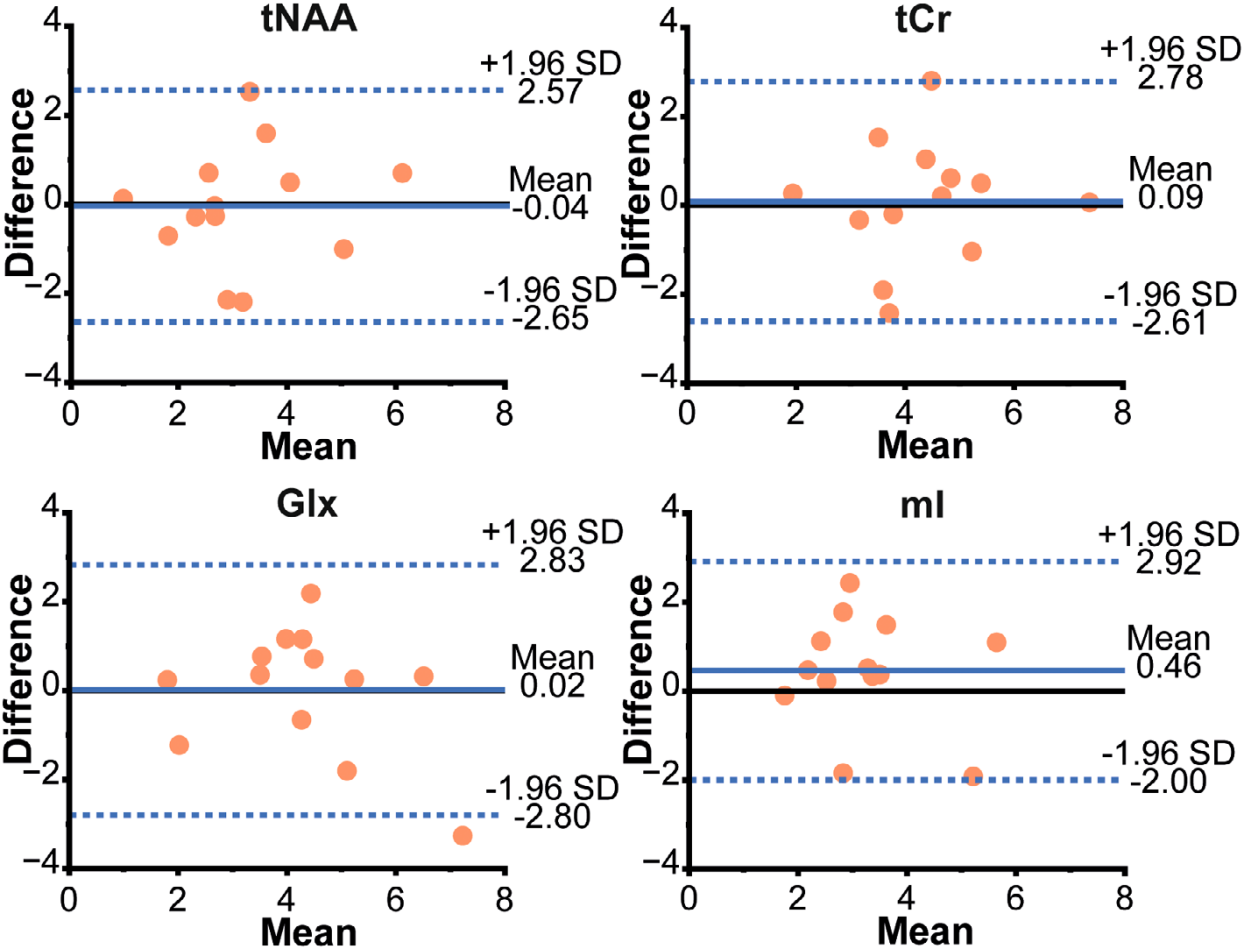
Bland–Altman plots illustrating agreement between two sessions (baseline and after 72 hours of fasting) for the concentrations of total NAA, total Cr, Glx and myo‐inositol in tumor tissue. tNAA = total NAA; tCr = total Cr; mI = myo‐inositol.

At baseline, besides Lac, Cho concentration was significantly higher in tumor compared to NAWM (1.90 ± 0.65 mM to 1.57 ± 0.48 mM) while significantly lower NAA and Cr concentrations were observed in tumor compared to NAWM. No significant differences were observed in the concentrations of metabolites that are not expected to change during the intervention. These metabolites were total NAA (*P* = 0.89, *P* = 0.64), total Cr (*P* = 0.69, *P* = 0.45), Glx (*P* = 0.69, *P* = 0.94), and myo‐inositol (*P* = 0.15, *P* = 0.22) in tumor (first *P*‐value) and NAWM (second *P*‐value). Further, Bland–Altman plots showed narrow limits of agreement (1.96 SD) between sessions for the concentration of metabolites in tumor with the absolute bias of 0.04 mM for total NAA concentration; 0.09 mM for total Cr; 0.02 mM for Glx and 0.46 mM for myo‐inositol (Fig. 4).

### Reproducibility of ^31^P Spectral Fitting and Quality

**FIGURE 5:**
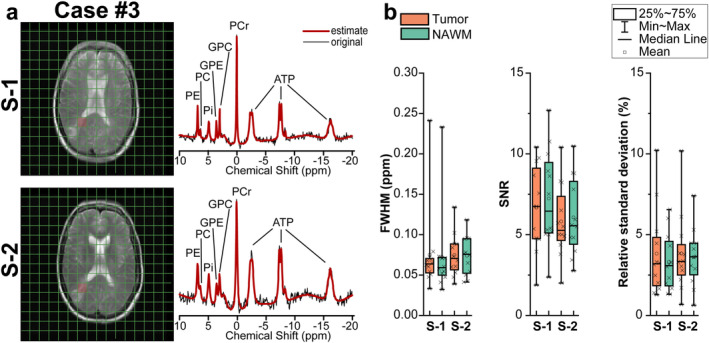
(**a**) Representative ^31^P CSI spectra for tumor tissue at baseline (S‐1) and after 72 hours of fasting (S‐2) at 3 T. The original signal is presented in black, and the AMARES spectral fit is in red. The positions of voxels on T2W images corresponding to the presented spectra are highlighted with red boxes. (**b**) Box plots of AMARES estimated metabolite linewidths and SNR of the ^31^P spectra, and relative SD of spectral fitting for phosphocreatine peak from the tumor and NAWM at baseline (S‐1) and after 72 hours of fasting (S‐2). Box‐and‐whisker plots are presented with minimum, maximum, interquartile ranges (25th and 75th percentiles), and median. PCr = phosphocreatine; PC = phosphocholine; PE = phosphoethanolamine; GPE = glycerophosphoethanolamine; GPC = glycerophosphocholine; ATP = adenosine triphosphate; Pi = inorganic phosphate.

The calculated spectral and fitting‐quality parameters (i.e., FWHM, SNR and relative SD of spectral fitting for phosphocreatine peak) are summarized in Fig. 5. The FWHM values extracted from both tumor and NAWM spectra were consistent between the two sessions (S‐1 vs. S‐2; *P* = 0.13, *P* = 0.15, from tumor and NAWM spectra, respectively). In addition, there were no significant differences in terms of SNR (*P* = 0.2) and relative SD of spectral fitting for phosphocreatine peak (*P* = 0.97) between sessions.

At baseline, pH value and PME/PDE ratio in tumor were significantly higher compared to NAWM (7.08 ± 0.05 to 7.03 ± 0.01; 0.71 ± 0.41 to 0.51 ± 0.16). The ATP/PCr ratio was not significantly different between tumor and NAWM (0.69 ± 0.08 to 0.67 ± 0.08, *P* = 0.47). In contrast to the ^1^H MRS data, there is no prior hypothesis for not affected metabolites during the intervention. Thus, the test–retest analysis of metabolite intensities in ^31^P MRS measurements is not reported here.

### Ketone Body Detection Using ^1^H sLASER CSI

As particularly seen in the tumor spectrum after the dietary intervention (S‐2; Fig. 2), the inverse doublet peak of β‐OHB at 1.19 ppm was well‐resolved and distinguishable from the Lac peak at 1.31 ppm. While Ace arose at 2.2 ppm as a singlet, AcAc peaks appeared at 2.27 and 3.34 ppm. In the spectra of the NAWM, flat baselines were observed, as expected from the measurements with long‐TE, due to the rapid decay of macromolecule and lipid signals. However, in tumor spectra, a slight baseline distortion was observed in the 3–4 ppm range because of insufficient water suppression in heterogeneous tumor tissue with a large necrotic area in the proximity of the voxel location. Nevertheless, baseline modulation was well handled by LCModel's baseline correction algorithm, allowing spectral fitting.

**FIGURE 6:**
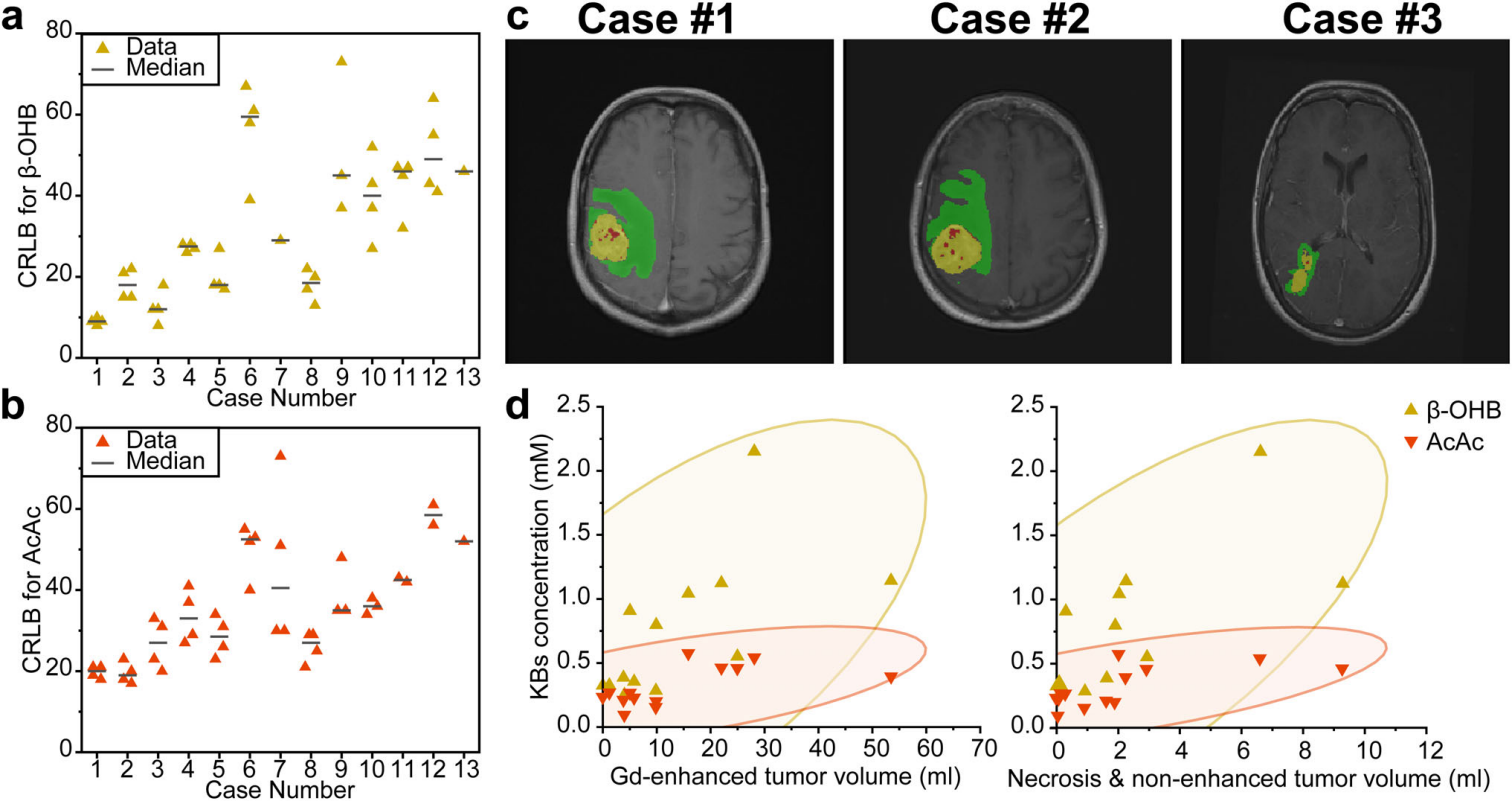
(**a**, **b**) CRLB values for β‐OHB and AcAc spectral fitting obtained from selected tumor voxels of 13 patients. The CRLBs of more than 80% were not shown here since they were not included in the statistical analysis. (**c**) Tumor segmentation results on three patients with high blood β‐OHB levels (>4 mmol/L) and KBs' accumulation in tumor. The results are overlaid on post‐contrast T1W images. Each color indicates a tumor class: red represents necrosis/non‐contrast‐enhanced tumor, green edema and yellow contrast‐enhanced tumor. (**d**) Spearman's correlation analysis between KBs' accumulation in tumor after 72‐hour‐fasting and existing tumor volume. The ellipses represent a 95% confidence interval. The concentrations of β‐OHB and AcAc were significantly correlated with necrosis/non‐contrast‐enhanced tumor volume (*ρ* = 0.81 and *ρ* = 0.76) and the volume of contrast‐enhanced tumor (*ρ* = 0.76 and *ρ* = 0.56).

Furthermore, β‐OHB and AcAc were detected with less than ~50% of CRLBs in tumor of all patients who completed a 72‐hour fasting cycle (Fig. 6a,b). The third KB, Ace, however, was observed only in 7/13 patients' tumor volume.

Remarkably, β‐OHB and AcAc were not only detected in the volume of the contrast‐enhanced tumor but also in the necrotic/non‐contrast‐enhanced tumor core. The concentrations of β‐OHB and AcAc were significantly correlated with the volume of contrast‐enhanced tumor (*ρ* = 0.76 and *ρ* = 0.56, respectively) and the necrosis/non‐contrast‐enhanced tumor core (*ρ* = 0.81 and *ρ* = 0.76, respectively). Examples of tumor segmentations and results of the correlation analyses are shown in Fig. 6c,d.

**FIGURE 7:**
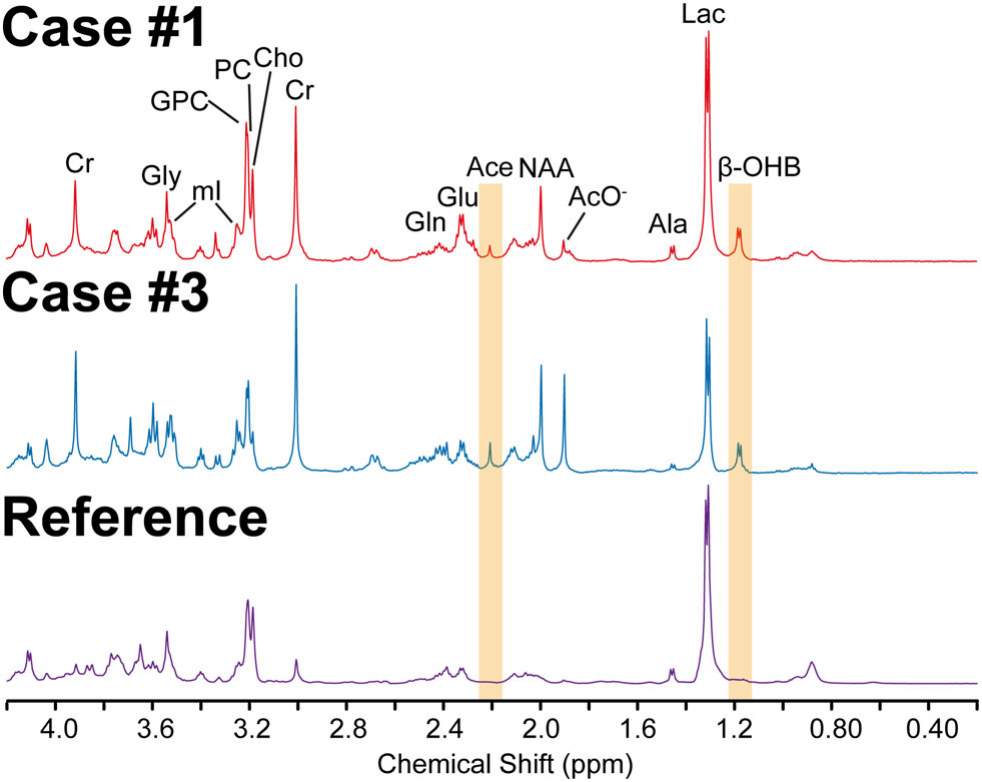
High resolution proton spectra of necrotic tissues extracted from Case #1 and #3 after 72 hours of fasting. A reference spectrum was obtained from a patient who did not follow the fasting intervention. Peaks of ketone bodies (i.e., Ace and β‐OHB) are highlighted with orange boxes. Gly = glycine; mI = myo‐inositol; GPC = glycerophosphocholine; PC = phosphocholine; Glu = glutamate; Gln = glutamine; AcO‐ = acetate; Ala = alanine.

The accumulation of KBs was also demonstrated by ex‐vivo ^1^H HR‐MAS measurements of necrotic tissues extracted after a 72‐hour fasting cycle (Fig. 7). In the spectra, the doublet of β‐OHB and the singlet of Ace appeared at 1.19 and 2.2 ppm, respectively. However, AcAc peaks were not evident which might be a result of its spontaneous (non‐enzymatic) decarboxylation to Ace in the extracted tissue during the storage.

**FIGURE 8:**
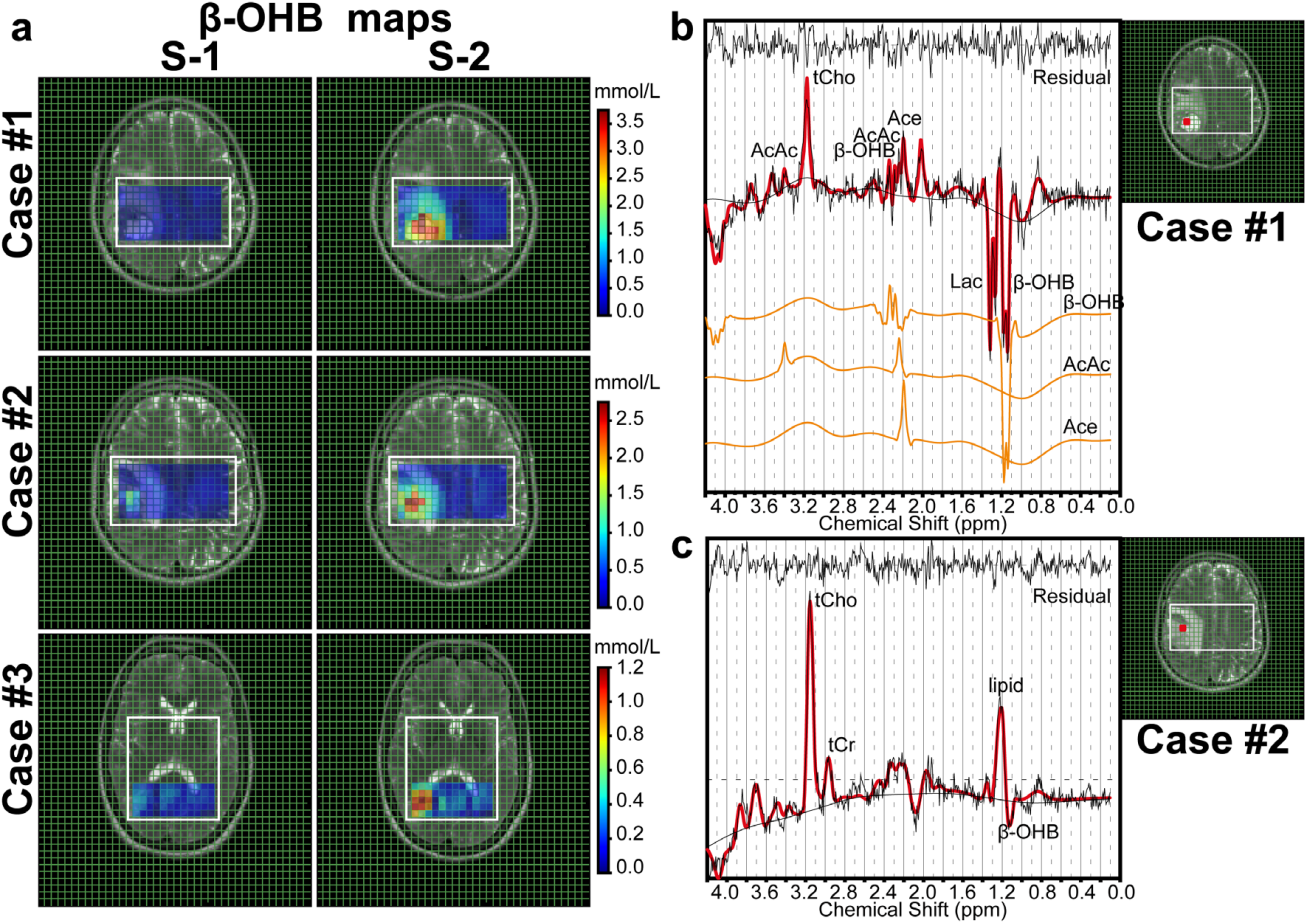
(**a**) β‐OHB maps registered on T2W images of three patients with high blood β‐OHB levels (>4 mmol/L) obtained from ^1^H sLASER CSI with TE of 144 msec measurement at baseline (left, S‐1) and after 72 hours of fasting (right, S‐2). (**b**, **c**) In vivo ^1^H CSI spectra of two patients obtained after 72 hours of fasting from the necrotic core of a glioblastoma. (b) The peaks that originated from KBs (β‐OHB, Ace, and AcAc) were observed with a substantially high amplitude and presented with individual fitting lines. (c) The β‐OHB peak was observed clearly despite elevated lipid levels in the spectra associated with necrosis and membrane breakdown. tCho = total Cho; tCr = total Cr.

The calculated β‐OHB concentration for each voxel was used to generate β‐OHB maps. These maps are shown in Fig. 8a for three patients with high blood β‐OHB levels (>4 mmol/L) after 72 hours of fasting. An increase in β‐OHB concentrations compared to baseline was found in the contrast‐enhanced tumor area and the necrosis/non‐enhancing tumor core (Fig. 8b). The inverse spectral pattern of β‐OHB as well as Lac allowed us to distinguish both from elevated lipid peaks (associated with membrane breakdown in necrotic glioma regions) arising in the 0.9–1.3 ppm range as shown in Fig. 8c.

## Discussion

We reported on the design of a dedicated, multi‐voxel, multi‐nuclei (^1^H/^31^P) MRSI protocol with fully automated segmentations of glioma sub‐regions for monitoring fasting‐induced changes. The goal was to account for key metabolites related to energy metabolism, tumor heterogeneity, and normal‐appearing brain regions.

We propose the use of a ^1^H sLASER CSI sequence at a TE of 144 msec for the detection of KBs and lactate at clinical field strengths such as 3 T. The reduced signal intensity of macromolecules and lipids and the inverted spectral patterns of β‐OHB and Lac at the suggested TE are particularly beneficial in tumor investigations where macromolecule and lipid profiles can vary substantially.^13^ As shown in our phantom studies, the commonly low signal intensity of Lac and β‐OHB obtained from the PRESS sequence at a TE of 144 msec is the result of a high chemical‐shift displacement error which causes anomalous J‐modulation.^15^ The use of the sLASER localization technique can reduce this chemical‐shift displacement error, allowing us to overcome the previously reported limitations in the use of ^1^H CSI at moderately long TEs and 3 T.

Due to the J‐evolution of J‐coupled metabolites at long TEs, the correct simulation of the LCModel basis set for the respective spectroscopic sequence is crucial for accurate spectral fitting and, therefore, for metabolite quantification. We carefully investigated spectral patterns of β‐OHB and Lac for various TEs. The good agreement between the simulated spectra and phantom measurements forms a prerequisite for the reliable in vivo quantification of brain metabolites.

We then demonstrated the feasibility, reliability, and repeatability of the suggested protocol in the context of a clinical study aimed at gaining a better understanding of diet‐induced changes in tumor tissue on an individual patient/tumor level. Our fitting/quality measures (^1^H MRS measurements: FWHM, SNR, CRLBs; ^31^P MRS measurements: FWHM, SNR, relative SD of spectral fitting) were favorable and highly consistent between both sessions. We demonstrated that intra‐subject reproducibility was maintained for metabolites that were not expected to change during the intervention with 72 hours between scans. In addition, the differences in ^1^H and ^31^P metabolite levels between tumor and NAWM at baseline measurements are consistent with previously published data.^19^

Obtaining well‐resolved spectra with sufficient linewidth allowed us to distinguish peaks in close proximity such as those of β‐OHB (1.19 ppm) and Lac (1.31 ppm). The chemical shift difference between the Ace peak (2.22 ppm) and the AcAc peak (2.27 ppm) corresponds to 6.4 Hz at 3 T, which exceeds the mean FWHM (5.7 Hz) obtained in this study. Furthermore, with the second peak of AcAc at 3.43 ppm we were able to discriminate between these two metabolite signals.

We found significantly elevated β‐OHB and AcAc in the volume of the contrast‐enhanced tumor and in the necrotic/non‐contrast‐enhanced tumor core of all patients who completed a 72‐hour fasting cycle. Further, we validated the accumulation of KBs in necrotic tumor tissue using ex‐vivo HR‐MAS NMR. The correlation between the segmentation volumes and KB concentrations led us to consider that the high concentrations, especially of β‐OHB, may be a result of neovascularization, the blood–brain barrier compromise, and no utilization of KBs in necrosis. Additional effects of metabolic plasticity on an individual patient/tumor level, taking intra‐tumoral heterogeneity into account, are currently being evaluated as part of the ERGO3 trial and will be reported at a later date. The third KB, Ace, was also detected in tumor tissues, but to a lesser extent (7/13 patients). Since Ace is formed from spontaneous decarboxylation of acetoacetate, its levels are much lower than those of the other two types of KBs. This lower concentration compared to β‐OHB might explain its lower detection rate at long TE.^35^, ^36^, ^37^

### Limitations

The major limitation of our study is the small sample size. Due to the lack of MRSI data for all patients and the exclusion of several MRSI data sets due to poor spectral quality as a result of unfavorable tumor location (temporal lobe), the number of complete MRSI data sets available for analysis was further reduced.

In long‐TE MR spectroscopic studies, the T_2_ decay of metabolite signals becomes a more relevant factor in metabolite quantification and should be taken into account in the calculation of each metabolite's concentration. While T_1_ and T_2_ relaxation times of standard metabolites in NAWM have been reported in the literature,^28^, ^29^, ^30^ and there is no reported relaxation time for KBs. Therefore, in this study, the T_1_ and T_2_ relaxation times of NAA were used for T_1_ and T_2_ relaxation correction of the concentration of KBs. In addition, T_2_ relaxation times of metabolites may differ in lesion and NAWM, and this difference has a higher impact on signal intensities in long‐TE measurements. Since T_2_ relaxation time values of metabolites in brain tumors are not consistent throughout the literature,^30^, ^38^ we used the same values for the quantification of metabolites in both tumor area and contralateral NAWM.

In general, the limitations of ^31^P MR spectroscopy originate from low SNR at clinical field strengths (1.5 T to 3 T). A large voxel size and a small matrix size were used to keep acquisition time tolerable. However, large voxels together with the poor point pread function entail partial volume effects which severely affect calculated metabolite concentrations from local lesions like tumors. This limitation can be addressed by speeding up data acquisition using accelerated k‐space trajectories, advanced reconstruction approaches, and compressed sensing methods.^39^, ^40^

## Conclusion

In vivo MRS including ^1^H sLASER with long‐TE and 3D ^31^P CSI may be used at clinical field strengths for monitoring diet‐induced changes in brain tumor tissue. The integration of multiparametric brain tumor segmentation with MRSI could facilitate the understanding of altered tumor metabolism under intervention despite intratumoral heterogeneity.

## Supporting information


**Data S1:** Supporting Information.
