# Supplementary material for: 2D 1H sLASER Long‐TE and 3D 31P Chemical Shift Imaging at 3 T for Monitoring Fasting‐Induced Changes in Brain Tumor Tissue
Source: J Magn Reson Imaging. 2024 May 9;61(1):426–38. doi: 10.1002/jmri.29422 (PMC11645487; doi:10.1002/jmri.29422)
Supplement: Supplementary file 1 — Data S1: Supporting Information. [file JMRI-61-426-s001.pdf]

Supplementary Information: 2D ^{1}H sLASER long-TE and 3D ^{31}P chemical shift imaging at 3T for monitoring fasting-induced changes in brain tumor tissue

Quantification of ^{1}H-MRS detectable Metabolites

Metabolite quantification was performed taking B1 inhomogeneity into account. Tissue water content was detected by the 2D ^{1}H FID CSI pulse sequence without performing water suppression. Metabolite concentrations in tissue water were calculated as described previously in Ref.[1] Metabolite signal intensities were corrected for T_1 and T_2 relaxation.[2–4] For relaxation correction of β-OHB, Ace, and AcAc signals, T_1 and T_2 relaxation times given for NAA in the literature were used.

After correction of metabolite signal intensities for T_1 and T_2 relaxation, B1 inhomogeneity was taken into account by division with $\sin\alpha$, where α, the real flip angle at the position of the voxel, was calculated from the B1 maps.[5] A receive profile of the coil (RP_{coil}) can be determined by

$$RP_{coil} = \frac{S}{\sin\alpha \times C \times V_{MRSI}},$$

where S represents the measured signal of a substance at concentration C and V_{MRSI} the MRSI voxel size. The metabolite concentration C_{met} acquired following a 90° flip angle can be calculated by

$$C_{met} = \frac{S_{met}}{RP_{coil} \times V_{MRSI}^{met}},$$

with S_{met} representing the relaxation corrected metabolite signals and V_{MRSI}^{met} for the voxel size in the respective sequence.

RP_{coil} at the slice of the ^{1}H MRSI data can be obtained from the FID MRSI data of water with

$$S_{wat} = \frac{S_{wat}^{FID-CSI}}{\sin\alpha}$$

where $S_{wat}^{FID-CSI}$ represents the water signal intensity, while the term $\sin\alpha$ takes into account the effect of B1 inhomogeneity ($B1^+$) and the 2° flip angle. Relaxation correction was not performed for water signals due to the small flip angle and the short delay between excitation and acquisition.

Using the following equation,

$$RP_{coil} = \frac{S_{wat}^{FID-CSI}}{\sin\alpha \times C_{wat} \times V_{MRSI}^{wat}},$$

where C_{wat} represents the concentration of water and V_{MRSI}^{wat} is the MRSI voxel size in FID-MRSI sequence, lastly, C_{met} can be calculated as follows:

$$C_{met} = \frac{\sin\alpha \times S_{met} \times V_{MRSI}^{wat} \times C_{wat}}{S_{wat}^{FID-CSI} \times V_{MRSI}^{met}}.$$

Since the voxels were positioned either in tumor or contralateral normal appearing white matter (NAWM), the concentrations of water in the voxels of interest were assumed as

$$C_{wat}^{WM} = 0.7 * 55.5\, {}^{mol}/_{L} = 38.85\, M.$$

^{1}H sLASER single voxel spectroscopy at 144 ms for β-OHB detection

As mentioned in the main text, in the measurements of patients with the tumor located in the temporal lobe, B0 static field homogeneity achieved using a 3D B0 field map for shimming (GRE-SHIM) was insufficient to obtain high-quality spectra from the tumor and contralateral normal-appearing brain tissue. In these cases, single voxel sLASER spectroscopy (SVS)[6-7] measurements with a TE of 144 ms were performed placing the voxel first at the tumor area and then the normal-appearing brain tissue on the contralateral hemisphere, anatomically corresponding to the selected tumor area. An example data set acquired from a glioma patient after 72 hours of fasting is demonstrated in **Supplementary Figure 1.** LCModel estimated SNR and linewidth values were 8 and 0.063 ppm for the tumor spectrum, and 7 and 0.048 ppm for the normal-appearing brain tissue spectrum, respectively. Even though high-quality spectra were obtained with reliable β-OHB detection (CRLB = 26%) using sLASER SVS sequence at TE of 144 ms from the temporal lobe, due to the lack of the complete data set, these measurements were excluded from the statistical analysis presented in the main text.

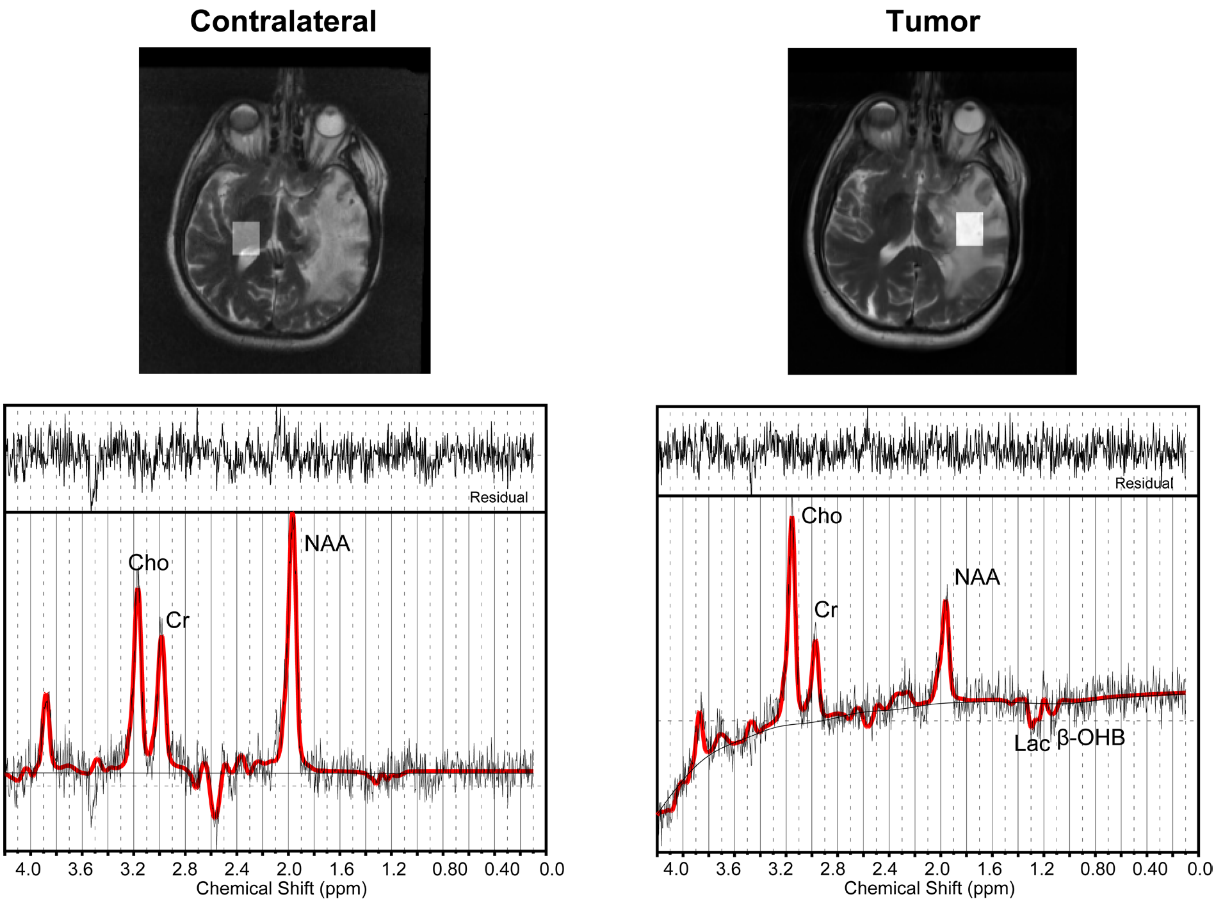

Supplementary Figure 1. An example analysis of ^{1}H MRS data acquired after 72 hours of fasting using LCModel with simulated basis data for KBs and standard metabolites. Boxes indicate voxel positioning on T2-weighted imaging. The original signal is presented in black, and the LCModel fit in red. The peaks originating from key metabolites including NAA, Cr, Cho, Lac, β-OHB are assigned.

MRSinMRS checklist

the Minimum Reporting Standards for in vivo Magnetic Resonance Spectroscopy (MRSinMRS) checklist can be found in Table 1.

Supplementary Table 1. MRSinMRS checklist for our multi-sequence MRS protocol.

Site (Name or Number)				
1. Hardware				
a. Field strength [T]	3 T	3 T	3 T	3 T
b. Manufacturer	Siemens	Siemens	Siemens	Siemens
c. Model (software version if available)	Prisma (VE11C)	Prisma (VE11C)	Prisma (VE11C)	Prisma (VE11C)

d. RF coils: nuclei (transmit/ receive), number of channels, type, body part	20 ch ^{1}H head coil	20 ch ^{1}H head coil	20 ch ^{1}H head coil	a double-tuned $^{1}H/^{31}P$ volume head coil
e. Additional hardware	N/A	N/A	N/A	N/A
2. Acquisition				
a. Pulse sequence	2D ^{1}H Semi-LASER CSI (vendor-based)	^{1}H Semi-LASER SVS	2D ^{1}H FID CSI (vendor-based)	3D ^{31}P FID CSI (vendor-based)
b. Volume of Interest (VOI) locations	Patients: tumor and contralateral	Patients: tumor and contralateral	Patients: tumor and contralateral	Patients: tumor and contralateral
c. Nominal VOI size [cm^3, mm^3]	Adjusted according to tumor volume with a slice thickness of 12 mm	20 x 20 x (15-20) mm^3	Adjusted according to tumor volume with a slice thickness of 25 mm	Adjusted according to tumor volume with a slice thickness of 25 mm
d. Repetition Time (TR), Echo Time (TE) [ms, s]	TR = 2000 ms, TE = 144 ms	TR = 2000 ms, TE = 144 ms	TR = 200 ms, delay = 2.3 ms	TR = 2000 ms, delay = 2.3 ms
e. Total number of Excitations or acquisitions per spectrum<br><br>In time series for kinetic studies<br><br>i. Number of Averaged spectra (NA) per time-point<br>ii. Averaging method (e.g. block-wise or moving average)<br>iii. Total number of spectra (acquired / in time-series)	2	128	1	10
f. Additional sequence parameters<br><br>(spectral width in Hz, number of spectral points, frequency offsets)<br><br>If STEAM:, Mixing Time (TM)	2000 Hz, 1024 points<br><br>delta frequency = −2.7 ppm<br><br>flip angle = 90°<br><br>2D: 240 × 240 × 12 mm3 FOV; matrix size 20 x 20 interpolated to 40 x 40; no acceleration factor;	2000 Hz, 2048 points<br><br>delta frequency = −2.7 ppm<br><br>flip angle = 90°<br><br>Excite pulse duration = 8960 µs	5000 Hz, 512 points<br><br>delta frequency = 0 ppm<br><br>flip angle = 2°<br><br>2D: 240 × 240 × 25 mm3 FOV; matrix size 20 x	2000 Hz, 1024 points<br><br>delta frequency = 0 ppm<br><br>flip angle = 60°<br><br>decoupling type: WALTZ4

If MRSI: 2D or 3D, FOV in all directions, matrix size, acceleration factors, sampling method	weighted distribution sampling; weighted distribution sampling	Refocus pulse duration: 8960 µs	20 interpolated to 40 x 40; no acceleration factor	decoupling duration: 1 ms<br><br>DC total duration: 30%<br><br>3D: 240 × 240 × 200 mm3 FOV; matrix size 8 x 8 x 8 interpolated to 16 x 16 x 16; no acceleration factor; weighted distribution sampling
g. Water Suppression Method	CHESS	VAPOR	None	N/A
h. Shimming Method, reference peak, and thresholds for "acceptance of shim" chosen	Automated 3D B0 field mapping technique (GRE-SHIM for brain)	Automated 3D B0 field mapping technique (GRE-SHIM for brain)	Automated 3D B0 field mapping technique (GRE-SHIM for brain)	Automated 3D B0 field mapping technique (GRE-SHIM for brain)
i. Triggering or motion correction method<br><br>(respiratory, peripheral, cardiac triggering, incl. device used and delays)	N/A	N/A	N/A	N/A
3. Data analysis methods and outputs				
a. Analysis software	LCmodel 6.2	LCmodel 6.2	jMRUI 6.0	jMRUI 6.0
b. Processing steps deviating from quoted reference or product	Basis set created using jMRUI 6.0 plug-in NMR-ScopeB	Basis set created using jMRUI 6.0 plug-in NMR-ScopeB	-	Gaussian line shapes, soft constraints for frequencies
c. Output measure<br><br>(e.g. absolute concentration, institutional units, ratio)Processing steps deviating from quoted reference or product	Ratios to water	Ratios to creatine	Used as water reference	Ratios to phosphocreatine and pH values

d. Quantification references and assumptions, fitting model assumptions	The basis set included spectra of 2-hydroxyglutarate, N-acetylaspartate, N-acetylaspartylglutamate, choline, creatine, γ-aminobutyric acid, glutamate, glutamine, myo-inositol, glutathione, glycine, alanine, lactate, β-hydroxybutirate, acetoacetate, and acetone. Macromolecules were not modelled.<br><br>LCModel sptype option has not been used.	The basis set included spectra of 2-hydroxyglutarate, N-acetylaspartate, N-acetylaspartylglutamate, choline, creatine, γ-aminobutyric acid, glutamate, glutamine, myo-inositol, glutathione, glycine, alanine, lactate, β-hydroxybutirate, acetoacetate, and acetone. Macromolecules were not modelled.<br><br>LCModel sptype option has not been used.	HLSVD fit of water signal	AMARES Gaussian lineshapes
4. Data Quality				
a. Reported variables<br><br>(SNR, Linewidth (with reference peaks))	SNR and linewidths are presented in Figure 3.	SNR and linewidths are presented in Supporting Information	SNR and linewidths of water reference not reported	SNR and linewidths are presented in Figure 5
b. Data exclusion criteria	LCModel SNR <3, LCModel FWHM >0.1 ppm.	LCModel SNR <3, LCModel FWHM > 0.1 ppm.	-	AMARES FWHM >0.25 ppm.
c. Quality measures of postprocessing Model fitting (e.g. CRLB, goodness of fit, SD of residual)	CRLB < 10% for total choline, total creatine, and total N-acetylaspartate (i.e., N-acetylaspartate + N-acetylaspartylglutamate); < 25% for Glx (i.e., glutamate + glutamine); < 40% for Lac and myo-inositol; > 80% for β-hydroxybutirate, acetoacetate, and acetone (Figure 3).	CRLB < 10% for total choline, total creatine, and total N-acetylaspartate (i.e., N-acetylaspartate + N-acetylaspartylglutamate); < 25% for Glx (i.e., glutamate + glutamine); < 40% for Lac and myo-inositol; > 80% for β-hydroxybutirate, acetoacetate, and acetone (Supporting Information).	Goodness of HLSVD fit of water signal	SD of residual > 15% PCr (Figure 5)
d. Sample Spectrum	Figure 2 and 3	Supporting Information		Figure 5

Acknowledgment:

The semi-LASER sequence was developed by Gülin Öz and Dinesh Deelchand and provided by the University of Minnesota under a C2P agreement.

References:

1. Silaidos C, Pilatus U, Grewal R, et al. Sex-associated differences in mitochondrial function in human peripheral blood mononuclear cells (PBMCs) and brain. *Biology of Sex Differences* 2018;9:34.

2. Mlynárik V, Gruber S, Moser E. Proton T 1 and T 2 relaxation times of human brain metabolites at 3 Tesla. *NMR in Biomedicine* 2001;14:325–31.

3. Landheer K, Sahgal A, Myrehaug S, et al. A rapid inversion technique for the measurement of longitudinal relaxation times of brain metabolites: application to lactate in high-grade gliomas at 3 T. *NMR in Biomedicine* 2016;29:1381–90.

4. Madan A, Ganji SK, An Z, et al. Proton T2 measurement and quantification of lactate in brain tumors by MRS at 3 Tesla in vivo. *Magnetic Resonance in Medicine* 2015;73:2094–9.

5. Volz S, Nöth U, Rotarska-Jagiela A, et al. A fast B1-mapping method for the correction and normalization of magnetization transfer ratio maps at 3 T. *NeuroImage* 2010;49:3015–26.

6. Deelchand DK, Berrington A, Noeske R, et al. Across-vendor standardization of semi-LASER for single-voxel MRS at 3T. *NMR Biomed* 2021;34:e4218.

7. Oz G, Tkáč I. Short-echo, single-shot, full-intensity proton magnetic resonance spectroscopy for neurochemical profiling at 4 T: validation in the cerebellum and brainstem. *Magn Reson Med* 2011;65:901–10.
